# Single-molecule FISH in *C. elegans* embryos reveals early embryonic expression dynamics of *par-2* , *lgl-1* and *chin-1* and possible differences between hyper-diverged strains

**DOI:** 10.17912/micropub.biology.000609

**Published:** 2022-07-20

**Authors:** Samiksha Kaul, Han Ting Chou, Seleipiri Charles, Guillaume Aubry, Hang Lu, Annalise B. Paaby

**Affiliations:** 1 School of Biological Sciences, Georgia Institute of Technology; 2 Wallace H. Coulter Department of Biomedical Engineering, Georgia Institute of Technology; 3 School of Chemical & Biomolecular Engineering, Georgia Institute of Technology; 4 Wallace H. Coulter Department of Biomedical Engineering and School of Chemical & Biomolecular Engineering, Georgia Institute of Technology

## Abstract

Wild *C. elegans* strains harbor natural variation in developmental pathways, but investigating these differences requires precise and well-powered phenotyping methods. Here we employ a microfluidics platform for single-molecule FISH to simultaneously visualize the transcripts of three genes in embryos of two distinct strains. We capture transcripts at high resolution by developmental stage in over one hundred embryos of each strain and observe wide-scale conservation of expression, but subtle differences in *par-2* and *chin-1* abundance and rate of change. As both genes reside in a genomic interval of hyper-divergence, these results may reflect consequences of pathway evolution over long timescales.

**Figure 1. Transcript abundance for par-2 , lgl-1 and chin-1 in embryos of the wild strain JU1088 and the wild-type reference strain N2, via single-molecule fluorescent in situ hybridization (smFISH):**
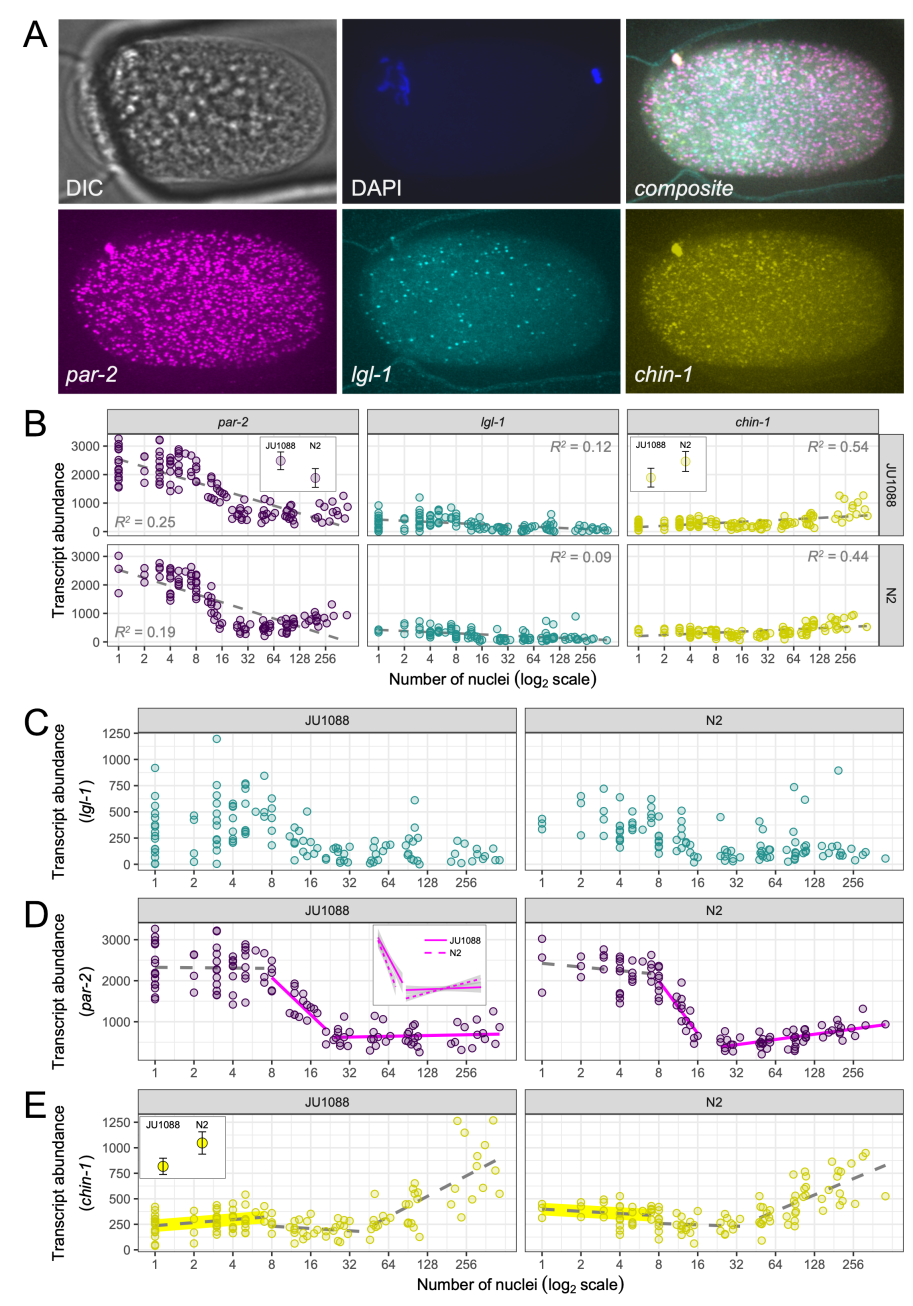
(A) Representative images of a single embryo of strain JU1088. The DIC channel shows the embryo within the microfluidics array, and the DNA stain in the DAPI channel indicates that this is an early-stage embryo shortly after fertilization. In the *par-2* , *lgl-1* , *chin-1* and composite channels, each spot indicates an individual RNA transcript. (B) Transcript counts for JU1088 (N=118) and N2 (N=105) embryos for the three genes, plotted over embryo stage given by number of nuclei. Each circle represents an individual embryo. The dashed lines show the estimated linear relationship between transcript abundance and embryonic stage. After controlling for stage, differences in adjusted transcript means suggest that *par-2* levels are higher in JU1088 ( *p* =0.009) and that *chin-1* levels are higher in N2 ( *p* =0.021*). Insets show adjusted means with 95% confidence. (C) - (E) The same data, now with free y-axis scales and transcript abundance regressed onto embryonic stage in separate intervals for *par-2* and *chin-1* . For *par-2* (D), solid lines over the second and third intervals indicate strain-wise differences in slope ( *p* =0.018* and *p* =0.001). Inset depicts regression lines with standard error in gray. For *chin-1* (E), the highlighted first segment indicates a difference in adjusted mean transcripts between the strains ( *p* =0.003). Inset shows adjusted means with 95% confidence. *Marginal significance; α _corrected_ =0.017 for all tests.

## Description

*C. elegans* embryogenesis is highly stereotyped, but developmental pathways harbor heritable variation among wild strains. Such variation has been revealed by variable penetrance of single-gene perturbations, indicating differences in pathway activity (Paaby et al. 2015). However, observing evidence of such “cryptic” variation at the cellular or molecular level is a challenge, in part because large sample sizes may be required to detect differences. In this report, we show that single-molecule fluorescent *in situ* hybridization (smFISH), applied simultaneously to multiple target genes on embryos arrayed in microfluidics chips, has the potential to capture wild-type dynamics in gene expression with high precision across embryonic stage. The dataset we present here is motivated by the observation that several critical genes in early embryogenesis are located in a region of chromosomal hyper-diversity in some wild strains.

The *C. elegans* embryo requires maternally-expressed genes to establish and maintain polarity, and in turn promote asymmetric cell division (Kemphues 2000). For example, localization of PAR ( *par* tition defective) proteins define the anterior and posterior domains of the one-cell embryo, including establishment of the posterior domain by PAR-1 and PAR-2 (Goldstein and Macara 2007). In addition to recruiting PAR-1 (Motegi et al. 2011; Ramanujam et al. 2018), PAR-2 works in a parallel pathway with LGL-1 and CHIN-1, two other proteins that localize to the posterior domain (Beatty et al. 2013). LGL-1 exhibits redundancy with PAR-2, and its overexpression can rescue *par-2* mutants (Beatty et al. 2010). CHIN-1 modulation of the Rho GTPase CDC-42 maintains cortical polarity of PAR proteins, and there is evidence that PAR-2 is required to recruit and stabilize CHIN-1 (Kumfer et al. 2010). As such, *par-2* , *lgl-1* and *chin-1* are three critical factors in the early embryo with a high degree of inter-activity.

Relative to the universal laboratory strain N2, the wild strain JU1088 harbors a 72kb region of hyper-diversity on chromosome III (Lee et al. 2021) that spans *par-2* and *chin-1* . Although most of the *C. elegans* genome exhibits relatively low polymorphism, punctuated regions show such hyper-diversity, potentially ancient relics from a non-selfing ancestor (Lee et al. 2021). The JU1088 alleles of *par-2* and *chin-1* exhibit an excess of single nucleotide polymorphisms and indels in both protein coding and UTR regions (83 total variants in *par-2* , 67 in *chin-1* ) but no high-impact mutations such as frameshifts or stop-gains (Cook et al. 2017), indicating that both genes remain under purifying selection and likely retain critical function. In contrast, *lgl-1* , which resides unlinked on the X chromosome outside of any hyper-diverged region, shows almost complete conservation between N2 and JU1088 (Cook et al. 2017).

As yet, functional consequences of hyper-diverged regions on genes in well-conserved pathways are unknown. Given the co-operation of *par-2* , *lgl-1* and *chin-1* in mediating embryonic polarity (Kumfer et al. 2010), the location of two of these genes in a hyper-diverged region in JU1088 (Lee et al. 2021), and previous evidence for heritable variation in embryonic pathway function between these strains (Paaby et al. 2015), we sought to assess native gene expression of these factors. We measured transcript abundance of the three genes simultaneously in embryos from just after fertilization to mid-late embryogenesis using smFISH, which visualizes individual RNA molecules via hybridization of fluorophore-tagged probes (Raj et al. 2008). Sufficient sample size was achieved by imaging embryos within microfluidic chips designed specifically for this application (Charles et al. 2021).

We imaged over 100 embryos for each strain (N=118 for JU1088, N=105 for N2) and quantified transcripts within each embryo using distinct fluorophores for *par-2* , *lgl-1* and *chin-1* (Figure 1A). Overall, *par-2* abundance was about 5.5 times greater than that of *lgl-1* and 4.0 times greater than that of *chin-1* , and patterns of expression appeared qualitatively similar between the strains (Figure 1B). These results suggest that expression of all three genes is well conserved, despite the location of *par-2* and *chin-1* in a hyper-diverged genomic region.

However, highly quantitative approaches have previously uncovered subtle, heritable differences in embryonic phenotypes that affect fitness (Farhadifar et al. 2015). To test for strain-wise differences in transcript abundance, we analyzed each gene separately and included embryonic stage as a covariate to control for potential differences in stage distribution between JU1088 and N2. For *par-2* and *chin-1* , the adjusted means were different between the strains: *par-2* abundance was higher in JU1088 by an estimate of 258.5 transcripts, or 20.4% ( *p* =0.009), and *chin-1* was lower in JU1088 by an estimate of 45.4 transcripts, or 12.0% ( *p* =0.021) (Table 1). Assuming a significance threshold of α=0.017 to correct for multiple tests, the difference at *chin-1* is only marginally significant. The adjusted means were not significantly different for *lgl-1* . For this gene, the regression of transcript counts onto embryonic stage was especially weak ( *R ^2^* =0.104 for whole model), owing in part to the fact that abundance did not change dramatically over developmental time in *lgl-1* (Figure 1C, Table 1). A direct test of means also showed no difference between strains for *lgl-1* ( *t* =0.824, df=218.62, *p* =0.411).

Both strains exhibit a drop in *par-2* levels just before the 8-cell stage, and a rise in *chin-1* levels after the 32-cell stage (Figure 1D-E). Changes in slope along the developmental time axis are likely due to transcript degradation and the maternal-zygotic transition; to test specific developmental phases for strain-wise differences, and to better control the effect of embryonic stage on transcript abundance, we repeated our analyses over subsetted intervals. For *par-2* , the first interval (1 to 7 cells) was no different between the strains, but the slopes were different in the second (8 to 23 cells) and third (24 to 460 cells) intervals ( *p* =0.018 and *p* =0.001; Figure 1D, Table 2). Heterogeneity between slopes precludes testing for differences in transcript means, as the effect of stage is uncontrolled. However, one interpretation is that *par-2* transcripts degrade faster and more completely and increase via zygotic expression more quickly in N2, and that this effect contributes to the apparent overall difference we first observed (Table 1). For *chin-1* , the slopes of all three intervals were no different between the strains (Figure 1E, Table 2), permitting comparison of transcript means. In the first interval (1 to 7 cells), the adjusted mean for N2 was 26.7% greater than that of JU1088 ( *p* =0.003), but there were no differences in the second (8 to 47 cells) or third (48 to 460 cells) intervals. This suggests that differences in *chin-1* levels between JU1088 and N2 embryos arise from differences in maternal inheritance, rather than transcript degradation or zygotic expression.

In conclusion, embryonic transcript levels and rates of change for the maternally inherited genes *par-2* , *lgl-1* and *chin-1* appear strongly conserved between JU1088 and N2. However, a granular analysis of the pseudo-timecourse revealed subtle differences in *par-2* and *lgl-1* between the strains. While the effect sizes are not immodest, the significance levels are marginal or close to marginal; we cannot rule out the potential influence of experimental or stochastic error, especially given that data were collected for a single chip for each strain. Assuming our observations reflect true biological differences, they may represent a phenotype under stabilizing selection and mutation-selection balance (Farhadifar et al. 2015). Consistent with this conclusion is the fact that only the hyper-diverged genes *par-2* and *chin-1* exhibited detectable differences. Though both genes harbor mutations in the 3' UTR, whether they affect maternal deposition, degradation, or zygotic expression is unknown. Future work may investigate potential consequences of the protein-coding mutations in *par-2* , *chin-1* , and other genes of conserved pathways in hyper-diverged regions.

**Table 1.** ANCOVA for *par-2* , *lgl-1* , and *chin-1* . Significance is indicated relative to family-wise α=0.017 following Dunn-Šidák correction.

**Table:** 

		Df	Sum Sq	Mean Sq	F	*p* -value	Significance
*par-2*	Stage	1	34824296	34824296	64.99	<0.0001	Sig
	Strain	1	3711225	3711225	6.93	0.009	Sig
	Residuals	220	117879599	535816			
*lgl-1*	Stage	1	1095080	1095080	27.20	<0.0001	Sig
	Strain	1	21117	21117	0.52	0.470	NS
	Residuals	220	8857608	40262			
*chin-1*	Stage	1	4681098	4681098	221.10	<0.0001	Sig
	Strain	1	114568	114568	5.411	0.021	Marginal
	Residuals	220	4657861	21172			

**Table 2.** Coefficient estimates following ANCOVA on segmented intervals of *par-2* and *chin-1* . Significance is indicated relative to family-wise α=0.017 following Dunn-Šidák correction.

**Table:** 

		Slope (rel to N2)			Adjusted mean (rel to N2)		
		Estimate	*p* -value	Significance	Estimate	*p* -value	Significance
*par-2*	Seg 1	-28.219	0.630	NS	61.137	0.801	NS
	Seg 2	-72.12	0.018	Marginal	NA	NA	NA
	Seg 3	1.3309	0.001	Sig	NA	NA	NA
*chin-1*	Seg 1	-24.329	0.062	NS	74.627	0.003	Sig
	Seg 2	0.7452	0.744	NS	23.6125	0.600	NS
	Seg 3	-0.2265	0.614	NS	59.7647	0.461	NS

## Methods

***C. elegans* Strains and Maintenance**

Strains used in this experiment include N2 and JU1088. Worms were grown on NGM plates at 20°C following standard protocol (Stiernagle 2006) for at least three generations without starving. Then, four days prior to the experiment, adult hermaphrodites were bleached and larvae were synchronized on empty plates prior to being transferred, in 4-hour intervals, onto plates seeded with OP50 bacteria. Samples were chosen for embryo collection by hand-selecting plates that were developmentally synchronous between the strains and included just-mature gravid animals in order to enrich for early stage embryos. For each strain, embryos were retrieved from a total of 12 plates and combined into a single sample for chip loading; all steps were performed simultaneously between the strains.

**smFISH Probe Design**

Probes for *par-2* , *lgl-1* and *chin-1* were designed using the Stellaris RNA FISH probe designer (Stellaris 2022a) and the CDS of the N2 reference genome on Wormbase (Harris et al. 2020). Candidate probes were then mapped to JU1088 sequence at each locus, derived from an independent long-read genome assembly (Chou et al. 2021) to ensure accuracy at the hyper-diverged region. Only probes at regions invariant between N2 and JU1088 were retained, and NCBI BLAST was used to ensure probe specificity to the gene of interest. Probe sets with reporter dyes Cal Fluor Red 590 ( *par-2* , 40 probes), FAM ( *lgl-1* , 40 probes), and Quasar 670 ( *chin-1* , 30 probes) were purchased from Biosearch Technologies.

**smFISH and Imaging**

Embryos were collected from day 1 adult hermaphrodites via bleaching (Stiernagle 2006) and prepared following modification of the Stellaris protocol for *C. elegans* (Stellaris 2022b). Specifically, embryos were fixed for 15min in 100-200µL fixation buffer in 1.5mL LoBind Protein Eppendorf tubes, then freeze-cracked in liquid nitrogen, washed twice with 1mL cold phosphate buffer saline, and rocked overnight in 1mL cold 70% ethanol at 4°C. Ethanol was removed by centrifugation and aspiration (all centrifugation was done at 900rcf for 2min) and embryos were then washed in 1mL wash buffer plus 0.06% tween-20. Following liquid removal by centrifugation and aspiration, 100µL of embryos were added to 100µL hybridization buffer plus probe. Probe sets were reconstituted following the manufacturer’s instruction and, following prior optimization, used at a concentration of 1.25µM in the hybridization buffer.

Embryos were hybridized at 37°C for 4hrs (no movement), then probes were removed by adding 1mL wash buffer plus 0.06% tween-20 directly to the tube and incubating at 37°C for 30min. Following centrifugation and aspiration, the wash was repeated once more, followed again by centrifugation and aspiration. Then, 100µL of non-hardening antifade medium with DAPI (VECTASHIELD, cat. no. H-1200-10 from Vector Laboratories) was added to 100µL of embryo solution. This final mixture was prepared for loading into chips by diluting up to 1mL with wash buffer. As described previously (Charles et al. 2021), PDMS-based microfluidics chips bonded to glass slides were used to distribute embryos in stationary arrays for high-throughput imaging on a motorized stage. A single chip was used for each strain. Images were obtained using a spinning disk confocal microscope (PerkinElmer UltraView VoX) equipped with a Hamamatsu C9100-23b back-thinned EM-CCD and a 100X oil immersion objective.

**Image Processing and Statistical Analysis**

Three-dimensional image stacks were collected using Volocity 3D visualization software (PerkinElmer) and exported as TIFF files. Image segmentation masks were applied manually in ImageJ (Schneider et al. 2012), then smFISH spots were quantified via Aro, a machine learning pipeline designed for single-molecule visualization in worm embryos (Wu and Rifkin 2015). The training sets for the random forest classifier were generated from multiple samples of each genetic background and treatment. To determine embryonic stage, chromosome clusters in the DAPI channel were counted manually in ImageJ (Schneider et al. 2012). Clusters typically span 19-25 slices in a z-stack; each new cluster was identified at the point when it emerged on a slice while cycling through the stack. For embryos with >100 chromosomes, the final count was determined by averaging counts done by two people. Example images for Figure 1 were exported from Volocity as grayscale image stacks, then merged, pseudo-colored for optimal colorblind discrimination, and brightness/contrast adjusted for publication in ImageJ (Schneider et al. 2012).

To evaluate whether transcript levels differ between the strains while accounting for changes by embryonic stage, we analyzed the data for each gene separately and included stage as a covariate (ANCOVA). This approach tests two hypotheses: whether the slopes of transcript counts regressed onto stage differ between the strains, and whether the adjusted transcript means differ between the strains. We first employed a model considering strain, stage and the interaction between them. For analyses over the entire developmental range, the interaction terms were non-significant in each case ( *p* =0.88 for *par-2* , *p* =0.68 for *lgl-1* , *p* =0.41 for *chin-1* ), implying homogeneity in slope and permitting inference about potential differences in adjusted means. We therefore dropped the interaction term from the model and analyzed the data again, to test only the hypothesis that (adjusted) transcript means differed between the strains after controlling for the effect of embryonic stage (results reported in Table 1). To further evaluate whether and how specific developmental intervals contributed to strain differences, we repeated the analysis on data subsetted by stage; intervals were identified by likely transitions in transcript degradation and zygotic expression. To correct for multiple testing, we compared *p* -values to a family-wise α following Dunn-Šidák correction, considering three comparisons in each analysis (three genes in the initial analysis, then three segments within each gene in the follow-up analyses). All analyses were performed in R (R Core Team 2021) and plotted with the packages *ggplot2* (Wickham 2016) and *viridis* (Garnier et al. 2021).

## Reagents

***C. elegans* strains**

**Table:** 

Strain	Genotype	Available from
N2	*Caenorhabditis elegans*	CGC
JU1088	*Caenorhabditis elegans*	CeNDR

**smFISH probes**

**Table:** 

Target gene	Fluorophore	Number of probes	Available from
*par-2*	Cal Fluor Red 590	40	Biosearch Technologies
*lgl-1*	FAM	40	Biosearch Technologies
*chin-1*	Quasar 670	30	Biosearch Technologies

**Solutions**

**Table:** 

Solution	Composition	Final Concentration
Fixation buffer	5mL 37% formaldehyde + 10mL 5X PBS (filtrated) + 35mL nuclease free water	3.7% formaldehyde in 1X phosphate buffered saline (PBS)
Wash buffer	5mL 20X SSC + 5mL deionized formamide + 40mL nuclease free water	10% formamide in 2X saline sodium citrate buffer (SSC)
Hybridization buffer	1g dextran sulfate + 10mL wash buffer	As wash buffer
